# Association of industry sponsorship and positive outcome in randomised controlled trials in general and abdominal surgery: protocol for a systematic review and empirical study

**DOI:** 10.1186/2046-4053-3-138

**Published:** 2014-11-27

**Authors:** Pascal Probst, Kathrin Grummich, Alexis Ulrich, Markus W Büchler, Phillip Knebel, Markus K Diener

**Affiliations:** University Hospital Heidelberg, Department of General, Visceral and Transplantation Surgery, University of Heidelberg, Im Neuenheimer Feld 110, 69120 Heidelberg, Germany; The Study Center of the German Surgical Society (SDGC), University of Heidelberg, Im Neuenheimer Feld 110, 69120 Heidelberg, Germany

**Keywords:** Industry bias, Industry sponsorship, General and abdominal surgery, Randomised controlled trial, Medical devices, Systematic review, Science study, Health care research

## Abstract

**Background:**

Industry sponsorship has been identified as a factor correlating with positive research findings in several fields of medical science. To date, the influence of industry sponsorship in general and abdominal surgery has not been fully studied. This protocol describes the rationale and planned conduct of a systematic review to determine the association between industry sponsorship and positive outcome in randomised controlled trials in general and abdominal surgery.

**Methods/design:**

A literature search in the Cochrane Library, MEDLINE and EMBASE and additional hand searches in relevant citations will be conducted. In order to cover all relevant areas of general and abdominal surgery, a new literature search strategy called multi-PICO search strategy (MPSS) has been developed. No language restriction will be applied. The search will be limited to publications between January 1985 and July 2014. Information on funding source, outcome, study characteristics and methodological quality will be extracted.

The association between industry sponsorship and positive outcome will be tested by a chi-squared test. A multivariate logistic regression analysis will be performed to control for possible confounders, such as number of study centres, multinational trials, methodological quality, journal impact factor and sample size.

**Discussion:**

This study was designed to clarify whether industry-sponsored trials report more positive outcomes than non-industry trials. It will be the first study to evaluate this topic in general and abdominal surgery. The findings of this study will enable surgical societies, in particular, to give advice about cooperation with the industry and disclosure of funding source based on empirical evidence.

**Systematic review registration:**

PROSPERO CRD42014010802

**Electronic supplementary material:**

The online version of this article (doi:10.1186/2046-4053-3-138) contains supplementary material, which is available to authorized users.

## Background

The debate about the presence and extent of inappropriate industry influence on medical professionals began in the 1980s [1, 2]. Shortly thereafter, a positive association between industry funding and positive research outcomes was first shown for pharmaceutical clinical trials [3]. This topic, referred to as industry bias, has meanwhile been studied in many different medical disciplines, and in 2012, a Cochrane review showed a relative risk of 1.32 (95% confidence interval 1.21–1.44) for industry-funded studies to report a positive outcome in a meta-analysis of 48 primary studies. One possible explanation for this difference between industry-funded trials and those with independent funding was found to be conclusions not justified by the study data. Other quality characteristics, such as risk of bias, did not differ between the two groups [4].

To our knowledge, 11 studies [5–15] have been published on the potential effect of industry sponsorship in various surgical disciplines. Five of these studies demonstrated a significant association of industry sponsorship and positive research outcome [6–8, 12, 13], and six studies did not show an association [5, 9–11, 14, 15] (Table 1). Only two, Lubowitz et al. for orthopaedic surgery and Sun et al. for surgery in otorhinolaryngology (ORL), searched trials systematically [9, 14]. The remaining nine studies selected their samples from one or more arbitrarily defined and mostly high-impact journals. This non-systematic approach represents the principal methodological limitation of existing studies into the effect of industry funding, as the sample might not be representative for the whole population of clinical trials in surgery.

**Table 1:** **Summary of 11 studies of the association between industry funding and positive research outcome across different surgical disciplines**

Author (reference)	Discipline	Search strategy	Investigated period	Included study type ( ***n*** )	Positive outcomes industry vs. independent
Leopold [8]	Orthopaedics	3 journals	12 months (1999–2000)	All (*n* = 301)	79% vs. 64% *p* = 0.0390
Ezzet [6]	Orthopaedics	3 journals, 2 congresses	12 months (2001–2002)	All (*n* = 173)	86% vs. 24% *p* < 0.0001
Bhandari [5]	Different fields of surgery	8 journals	18 months (1999–2001)	RCT (*n* = 87)	81% vs. 68% *p* = 0.4385
Shah [13]	Orthopaedics	1 journal	19 months (2002–2003)	All (*n* = 527)	73% vs. 44% *p* < 0.0001
Lynch [10]	Orthopaedics	1 journal (submitted manuscripts)	17 months (2004–2005)	All (*n* = 208)	74% vs. 70% *p* = 0.7070
Okike [12]	Orthopaedics	2 congresses	2001 + 2002	All (*n* = 494)	98% vs. 88% *p* = 0.0258
Lubowitz [9]	Orthopaedics	MEDLINE	Open–2005	CCT/RCT (*n* = 23)	100% vs. 86% *p* = 0.6637
Yao [15]	ORL	4 journals	60 months (2000–2005)	RCT (*n* = 202)	81% vs. 79% *p* = 0.8538
Khan [7]	Orthopaedics	5 journals	24 months (2002–2004)	RCT (*n* = 100)	85% vs. <45% *p* < 0.0001
Momeni [11]	Plastic surgery	3 journals	15 years (1990–2005)	CCT/RCT (*n* = 63)	74% vs. 64% *p* = 0.5900
Sun [14]	ORL	MEDLINE, EMBASE, CINAHL, CENTRAL	1960–2010	RCT (*n* = 118)	35% vs. 51% *p* = 0.2067

*CCT* controlled clinical trial.

General and abdominal surgery comprises a large field of different operations. Abdominal surgery involves operations on organs like stomach, liver, pancreas and gut. Whereas, general surgery involves an inhomogeneous spectrum of operations, e.g. surgery of the thyroid gland, hernias and proctology. Surgery is a field with high potential for innovation because of the constant development of new interventions, especially with regard to medical devices. Implementation of new interventions is commonly justified on the basis of clinical trials. Thus, any industry bias would have a relevant impact on surgical practice. This protocol describes the methods to perform a systematic literature search to find a representative sample of trials for a primary statistical analysis. The influence of industry sponsorship in general and abdominal surgery will be evaluated for the first time.

## Methods/design

### Research question

This study will firstly aim to determine whether there is an association between industry sponsorship and positive outcome in randomised controlled trials (RCTs) in the field of general and abdominal surgery.

Second, methodological differences between industry- and non-industry-funded RCT will be evaluated in order to explore potential sources of industry bias.

### Systematic literature search methodology

**Figure 1:**
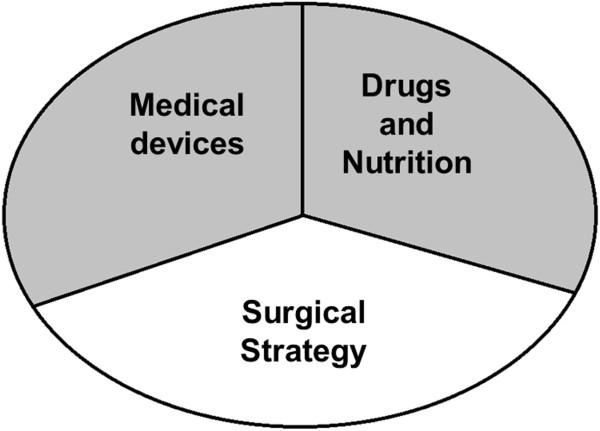
**Conceptual visualisation of existing RCT in surgery, divided into three major areas: medical devices, drugs/nutrition and surgical strategy.** The grey shading represents the study sample at risk for industry bias.

One of the major challenges of this study will be the identification of a representative sample of trials within the field of general and abdominal surgery that is potentially at risk for industry bias. Trials without intrinsic commercial interest will have to be excluded from the study sample (Figure 1). Consequently, trials evaluating surgical strategies such as the Shouldice vs. Bassini operation for repair of inguinal hernia will not be investigated as no commercial interest can be directly related to the efficacy of the experimental intervention. On the basis of a preliminary literature screening, trials dealing with medical devices and pharmacological and nutritional interventions represent the vast majority of research with potential industry involvement.

**Figure 2:**
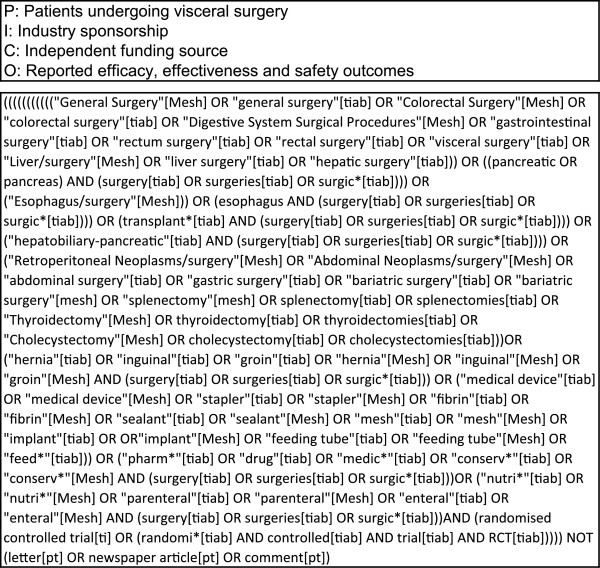
**Preliminary PICO question and holistic search strategy.**

**Figure 3:**
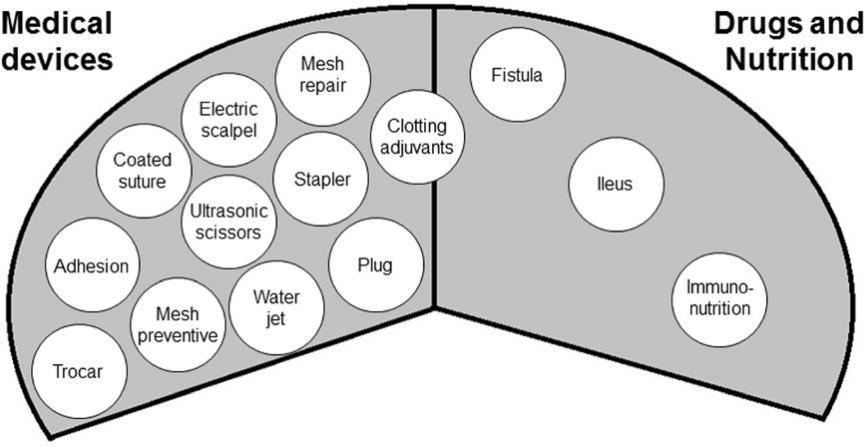
**On the basis of a preliminary literature screening, subfields with potential industrial background were recorded.** Trials from these subfields will be gathered according to a multi-PICO search strategy.

**Figure 4:**
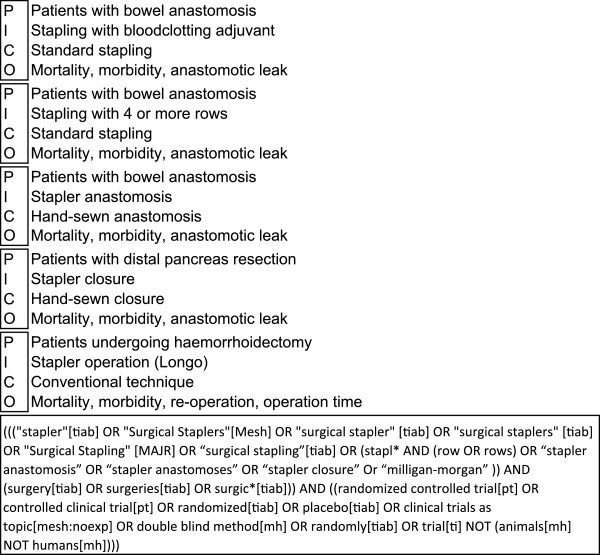
**Minor PICOs for the subfield Stapler.**

A research question was formulated according to the Participants, Interventions, Comparisons and Outcomes (PICO) model [16]. Figure 2 shows the preliminary PICO question with the search strategy. Due to the holistic approach, this search strategy was very unspecific, with precision below 3% on the abstract screening. Alteration of search terms did not improve precision substantially. However, abstracts identified by the preliminary search were screened for surgical interventions of interest and classified into 14 subfields (Figure 3). On this basis, a new form of comprehensive literature search was developed, the “multi-PICO search strategy” (MPSS):

–First, the preliminary PICO question served as the “master PICO”.
–Second, one or more specific questions called “minor PICOs” were created for every recorded subfield (e.g. Stapler, Figure 4).

Based on the minor PICOs, a systematic literature search will be performed independently by two reviewers following the recommendations of the Cochrane Collaboration [16]. The following databases will be searched: Cochrane Library, MEDLINE (via PubMed) and EMBASE. A search strategy based on a vocabulary thesaurus (MeSH or Emtree) in combination with text words will be used. Additionally, a hand search in relevant citations will be performed. The search will be limited to the period from January 1985 to July 2014, with the rationale that disclosure of funding source was not demanded before 1985. No language restrictions will be applied.

All details of the performed MPSS are displayed in Additional file 1. Archiving of MPSS followed the same rules as for a single-PICO search strategy [17].

The method of MPSS allowed the creation of a specific search for every subfield of investigation.

### Study selection

Articles gathered by the MPSS will be screened for eligibility according to the following criteria.

### Inclusion criteria

RCT assessing the efficacy or effectiveness of medical devices and perioperative pharmacological and nutritional interventions with direct relation to the surgical procedure in human patient populations in the field of general and abdominal surgery will be eligible.

### Exclusion criteria

RCT without funding information.

RCT assessing neurosurgical, urological, orthopaedic, dental, plastic, cardiothoracic, gynaecologic, dermal, vascular or paediatric surgery and otorhinolaryngological or endoscopic interventions.

Thus, a systematic search for surgical RCT in general and abdominal surgery with potential risk for industry bias will yield a broad and representative sample to answer the primary research question.

### Data extraction

The full data extraction sheet is displayed in Additional file 2. Here, only items evaluating the research question are defined (Table 2).

**Table 2:** **Extracted information to evaluate the research question**

Funding source	[Industry/independent]
Experimental intervention is reported to be superior to the control intervention	[Yes/no]
Exact *p* value of the primary endpoint	[*n*]
Year of publication	[*n*]
Impact factor of journal	[*n*]
Region	[National/multinational]
Number of study centres	[*n*]
Sample size	[*n*]
Concluded superiority without statisticalsignificance of primary endpoint	[Yes/no]
Risk of bias for primary endpoint according to Cochrane Collaboration’s tool for assessing risk of bias	[Low/high/unclear]

A trial will be classed as industry funded if any funding is explicitly stated, regardless of whether the funding took the form of direct financial support, supply of products for use in the study or the conduct of trial tasks, e.g. data analysis. Trials will be dichotomised according to whether the authors conclude the experimental intervention to be superior to the control intervention. The conclusions drawn by the authors will be compared to the data presented. A conclusion in favour of the experimental intervention based on non-significant differences between groups will be recorded. Risk of bias will be assessed according to the Cochrane Collaboration’s tool for assessing risk of bias [16]. Further study characteristics will be captured for multivariate analysis as stated in the “Statistical analysis” section below.

Data extraction will be performed by two reviewers independently for quality assurance purposes [18]. Discrepancies between the two reviewers will be resolved by a third reviewer, and a final extraction sheet will be determined for database entry. A database monitoring will be performed of 100% of data necessary to evaluate the primary endpoint and a randomly selected 20% of remaining data. Finally, the database will be closed and made available for statistical analysis.

### Statistical analysis

A primary statistical analysis will be performed to answer the primary research question regarding the association of industry sponsorship and positive outcome as well as the magnitude of this association. Therefore, trials will be divided into those funded by industry and those not funded by industry. Further, trials will be dichotomised according to whether or not the experimental intervention is reported to be superior to the control group. The chance that industry-funded trials report more positive outcomes is expressed as odds ratio (OR). The null hypothesis (H0) is that industry funding is not associated with a positive trial outcome. The alternative hypothesis (H1) is that industry funding is associated with a positive trial outcome. The significance of association will be tested by means of Fisher’s exact test if at least one value in the contingency table is 5 or below. Pearson’s chi-squared test with Yates’s correction will be used if the total sample size is 60 or less. In all other cases, significance of association will be tested using Pearson’s chi-squared test without Yates’s correction at a level of significance of 5%. Furthermore, a multivariate logistic regression with factors (multinational trials, methodological quality) and covariates (number of study centres, journal impact factor, sample size) will be conducted. An additional analysis will be performed for the three subgroups medical devices, pharmaceuticals and nutrition.

Moreover, by comparison of reported *p* values of primary endpoints from industry-funded and independently funded trials, a possible industry bias will be quantified. Student’s *t*-test or the Wilcoxon rank-sum test will be used for exact *p* values. If 20% of *p* values are not reported exactly, the *p* values will be classified and Fisher’s exact test or a chi-squared test will be performed.

Additional data extracted will be presented descriptively. Publication bias will be explored using a funnel plot separately for industry-sponsored trials and non-industry- sponsored trials. Statistical analysis will be performed with *R*[19].

## Discussion

Existing literature about association of industry sponsorship and positive outcome in surgery has major limitations due to the approach to primary trials as mentioned above. Therefore, the presence and extent of such association in randomised controlled trials in general and abdominal surgery remains unexplored.

In the case of the hypothesised association of industry sponsorship and positive outcome, this study will investigate by a multivariate statistical analysis whether industry involvement biases result via standard risk domains or if industry involvement is an independent source of bias as assumed by several studies [4, 20].

The conduct of this study is important, because the detection of an industry bias in surgery would have an impact on future research. The findings of this study will enable surgical societies, in particular, to give advice about cooperation with industry and disclosure of funding source based on empirical evidence.

## Authors’ information

PP is a surgical resident and holds an MSc in clinical trial management. KG is a methodological specialist and core member of the surgical systematic review group at the Study Center of the German Surgical Society. PK is a board certified surgeon and head of the surgical clinical trial unit. AU is chief consultant in the surgical department and head of surgical oncology. MWB is full professor of the department of general, visceral and transplantation surgery. MKD is a consultant in the surgical department and head of the Study Center of the German Surgical Society.

## Electronic supplementary material

Additional file 1: **Detailed MPSS.** Detailed information on the multi-PICO search strategy including search terms for MEDLINE. (PDF 93 KB)

Additional file 2: **Extraction sheet.** Standardised data collection form that will be used to extract data from included studies. (PDF 289 KB)

## Abbreviations

CCT: controlled clinical trial
MPSS: multi-PICO search strategy
ORL: otorhinolaryngology
PICO: Participants Interventions, Comparisons and Outcomes
RCT: Randomised controlled trials.
