# Supplementary material for: Association of industry sponsorship and positive outcome in randomised controlled trials in general and abdominal surgery: protocol for a systematic review and empirical study
Source: Syst Rev. 2014 Nov 27;3:138. doi: 10.1186/2046-4053-3-138 (PMC4280764; doi:10.1186/2046-4053-3-138)
Supplement: Supplementary file 1 — Additional file 1: Detailed MPSS. Detailed information on the multi-PICO search strategy including search terms for MEDLINE. (PDF 93 KB) [file 13643_2014_313_MOESM1_ESM.pdf]

<u>Multi-PICO Search Strategy (MPSS) of Association of industry sponsorship and positive outcome in randomised controlled trials in general and abdominal surgery</u>

1. **Master-PICO**

P: Patients undergoing visceral surgery
I: Industry sponsorship
C: Independent funding source
O: Reported efficacy, effectiveness and safety outcomes

2. **Minor-PICO**

<u>Medical devices</u>
Mesh Preventive

P: Patients undergoing abdominal surgery
I: Preventive mesh
C: Standard abdominal closure
O: Mortality, morbidity, incision hernia

P: Patients undergoing surgery with stoma placement
I: Preventive mesh placement
C: Standard operation
O: Mortality, morbidity, Operation time, stoma hernia

((((((„hernia prevention“ OR „hernia prophylaxis“ OR „hernia, Abdominal/prevention and control“ [Mesh] OR “Hernia, Ventral/prevention and control” [Mesh] OR “Hernia/prevention and control” [Mesh] OR “incisional hernia” OR “abdominal wall hernia” OR “incisional herniation”)) OR ("stoma" AND ("hernia"[MeSH Terms]OR hernia [tiab])))) AND (((“mesh placement” [tiab] OR “mesh implantation” [tiab] OR “prophylactic mesh” [tiab])) OR (mesh [tiab] AND prevent* [tiab])))) AND (randomized controlled trial [pt] OR random*)

Mesh Repair

P: Patients undergoing open hernia surgery
I: Glue fixation
C: Standard fixation
O: Mortality, morbidity, hernia recurrence

P: Patients undergoing open hernia surgery
I: Self-fixating mesh
C: Standard mesh
O: Mortality, morbidity, hernia recurrence

P: Patients undergoing hernia surgery
I: Bio tissue
C: Standard mesh
O: Mortality, morbidity, hernia recurrence

P: Patients undergoing groin hernia surgery
I: Plug meshes
C: Standard mesh
O: Mortality, morbidity, hernia recurrence

P: Patients undergoing laparoscopic groin hernia surgery
I: Preparation with a spacemaker
C: Standard laparoscopic preparation
O: Mortality, morbidity, Operation time

((((("Hernia, Abdominal/surgery"[Mesh] OR "Hernia, Ventral/surgery"[Mesh] OR "Hernia, Inguinal/surgery"[Mesh] OR “groin hernia” OR “incisional hernia” OR “abdominal wall hernia” OR “incisional herniation”)) OR ("stoma" AND ("hernia"[MeSH Terms]OR hernia [tiab]))) AND (glue[tiab] OR "mesh fixation" OR "self fixating mesh" OR grip [tiab] OR "absorbable mesh" OR Gore [tiab] OR "biologic mesh" OR "biological mesh" OR "bio tissue" OR "light weight mesh" OR plug [tiab] OR "Dissection Balloon" OR "balloon dissector" OR "balloon dissection"))) AND (randomized controlled trial [pt] OR random*)

Stapler

P: Patients with bowel anastomosis
I: Stapling with bloodclotting adjuvant
C: Standard stapling
O: Mortality, morbidity, anastomotic leak

P: Patients with bowel anastomosis
I: Stapling with 4 or more rows
C: Standard stapling
O: Mortality, morbidity, anastomotic leak

P: Patients with bowel anastomosis
I: Stapler anastomosis
C: Hand-sewn anastomosis
O: Mortality, morbidity, anastomotic leak

P: Patients with distal pancreas resection
I: Stapler closure
C: Hand-sewn closure
O: Mortality, morbidity, anastomotic leak

P: Patients undergoing haemorrhoidectomy
I: Stapler operation (Longo)
C: Conventional technique
O: Mortality, morbidity, re-operation, operation time

((("stapler"[tiab] OR "Surgical Staplers"[Mesh] OR "surgical stapler" [tiab] OR "surgical staplers" [tiab] OR "Surgical Stapling" [MAJR] OR “surgical stapling”[tiab] OR (stapl* AND (row OR rows) OR “stapler anastomosis” OR “stapler anastomoses” OR “stapler closure” Or “milligan-morgan”)) AND (surgery[tiab] OR surgeries[tiab] OR surgic*[tiab])) AND ((randomized controlled trial[pt] OR controlled clinical trial[pt] OR randomized[tiab] OR placebo[tiab] OR clinical trials as topic[mesh:noexp] OR double blind method[mh] OR randomly[tiab] OR trial[ti] NOT (animals[mh] NOT humans[mh])))

Ultrasonic scissors

P: Patients undergoing thyroidectomie
I: Ultrasonic scissors
C: Standard operation
O: Mortality, morbidity, operation time

P: Patients undergoing haemorrhoidectomy
I: Ultrasonic scissors
C: Conventional technique
O: Mortality, morbidity, re-operation, operation time

((((((thyroid) AND (surgery OR operation OR resection OR lobectomy)) OR (thyroidectomy OR thyroidectomies OR hemithyroidectomy OR hemithyroidectomies) OR (thyroidectomy[MeSH Terms])) AND (((vessel OR vascular) AND (sealing OR occlusion)) OR (hemostasis OR haemostasis OR devascularization OR diathermy OR "bleeding control") OR (Hemostatic Techniques[MeSH Terms])) AND ((conventional OR traditional OR monopolar OR ligation OR tying OR tie OR ties OR clamp OR clamps OR clamping OR clip OR clips OR knot OR knots) OR (sutureless OR device OR devices OR coagulator OR coagulation OR energized OR energised OR bipolar OR electrocoagulation OR electrothermal OR electrosealing OR ligasure OR thermostapler OR harmonic OR ultrasonic OR focus OR ultracision))))) AND (((randomized controlled trial [ptyp] OR random*)))

Water jet dissector

P: Patients undergoing liver dissection
I: Water jet dissection
C: Standard dissection
O: Mortality, morbidity, operation time

("water jet dissector" OR "water jet dissection" OR “hydro-jet” OR hydrojet OR hydrodissection OR "jet-cutter") AND random*

Trocar

P: Patients undergoing laparoscopic surgery
I: Mini-Trocars
C: Standard Trocars
O: Mortality, morbidity, incision hernia, cosmetic

((“mini trocar” [tiab] OR “mini trocars” [tiab] OR “mini-trocar” [tiab] OR “mini-trocars” [tiab] OR “mini instrument” [tiab] OR “mini instruments” [tiab] OR “mini-instrument” [tiab] OR “mini-instruments” [tiab] OR “mini-lap” [tiab] OR “mini lap” [tiab] OR “mini-laparoscopy” [tiab] OR “mini laparoscopy” [tiab] OR M-LC)) AND (randomized controlled trial [ptyp] OR random*)

P: Patients undergoing thyroid or parathyroid surgery
I: Mini invasive approach
C: Standard approach
O: Mortality, morbidity, operation time, cosmetic

((((“minimal-invasive surgery” [tiab] OR “minimal-invasive surgeries” [tiab] OR “mini invasive approach” [tiab] OR “mini invasive approaches” [tiab] OR “mini-invasive approach” [tiab] OR “mini-invasive approaches” [tiab])) AND (((thyroidectomy [Mesh] OR thyroidectom* [tiab] OR “thyroid gland resection” [tiab] OR “thyroid lobectomy” OR “thyroid lobectomies” OR "partial thyroidectomy" [tiab] OR “partial thyroidectomies” [tiab] OR "subtotal thyroidectomy" [tiab] OR “subtotal thyroidectomies” [tiab] OR hemithyroidectomy [tiab] OR hemithyroidectomies [tiab] OR “thyroid operation” [tiab] OR “thyroid operations” [tiab] OR “thyroid surgery” [tiab] OR “thyroid surgeries” [tiab])) OR (parathyreoidectom* [tiab] OR “parathyroid surgery” [tiab] OR “parathyroid surgeries” [tiab] OR “parathyroid resection” [tiab])))) AND ((randomized controlled trial [ptyp] OR random*))

Electric scalpel

P: Patients undergoing abdominal surgery
I: Skin incision with electric scalpel
C: Standard incision
O: Mortality, morbidity, surgical site infection, bleeding, incision hernia, cosmetic

P: Patients undergoing abdominal surgery
I: Organ preparation with electric scalpel
C: Standard preparation
O: Mortality, morbidity, surgical site infection, bleeding, incision hernia, cosmetic

((((((((“electric scalpel” [tiab] OR “electric scalpels” [tiab])) OR (electrosurg* [tiab] OR electrodissect* [tiab] OR electrocaut* [tiab] OR electrocoagul* [tiab])) OR (thermocaut* [tiab] OR thermocoagul* [tiab])) OR diathermy [tiab] OR bipolar [tiab])) AND ((surgery[tiab] OR surgeries[tiab] OR surgic*[tiab])))) AND (randomized controlled trial [ptyp] OR random*)

Plug

P: Patients undergoing anal fistula repair
I: Plug
C: Standard operation
O: Mortality, morbidity, fistula closure rate

("anal fistula" OR "Rectal Fistula"[Mesh] OR "perianal fistula" OR "fistula-in-ano") AND (plug OR "fistula plug") AND (randomized controlled trial [ptyp] OR random*)

Coated suture

P: Patients undergoing abdominal surgery
I: Coated suture
C: Standard abdominal closure
O: Mortality, morbidity, surgical site infection

(((("Sutures"[Mesh] OR (suture [tiab] OR sutures [tiab]))) AND ((coated OR impregnated)))) AND (randomized controlled trial [ptyp] OR random*)

Clotting adjuvants

P: Patients undergoing bile duct and liver surgery
I: Clotting adjuvant
C: Standard measures
O: Mortality, morbidity, bile leak

P: Patients undergoing pancreatic surgery
I: Clotting adjuvant
C: Standard measures
O: Mortality, morbidity, POPF

P: Patients undergoing abdominal surgery
I: Clotting adjuvant
C: Standard bleeding control
O: Mortality, morbidity, Reoperation/ Reintervention for bleeding

((((("tachocomb "[Substance Name]) OR (tachocomb[tw]) OR ("TachoSil "[Substance Name]) OR (TachoSil[tw]) OR ("Coated Materials, Biocompatible/therapeutic use"[Mesh]) OR ("Fibrin Tissue Adhesive/therapeutic use"[Mesh]) OR ("Fibrinogen/therapeutic use"[Mesh]) OR ("Thrombin/therapeutic use"[Mesh]) OR (Fibrin Tissue Adhesive[tiab]) OR (Fibrin Tissue[tiab]) OR (Fibrin Sealant*[tiab]) OR Collagen Fleece[tiab] OR Tissue Adhesives[tiab])) AND (((surgery[tiab] OR surgeries[tiab] OR surgic*[tiab]))))))) AND ((randomized controlled trial [ptyp] OR random*))

Adhesion prevention

P: Patients undergoing abdominal surgery
I: Adhesion prevention device
C: Placebo or nil
O: Mortality, morbidity, ileus, Adhesions seen by laparoscopy

(((((("icodextrin" [Supplementary Concept] OR icodextrin [tiab] OR "Seprafilm" [Supplementary Concept] OR sepracoat OR seprafilm OR "spraygel adhesion barrier" OR oxiplex-ap gel OR Oxiplex [Supplementary Concept] OR Adept [tiab])) OR ((((((((Interceed[tiab]) OR tc7[tiab]) OR sprayshield[tiab]) OR spraygel[tiab]) OR polyethylene glycol[tiab]) OR (Hyaluronic Acid/therapeutic use[mesh])) OR hyaluronic acid [tiab] OR Carboxymethylcellulose Sodium/therapeutic use[mesh] OR carboxymethylcellulose [tiab]) OR Polyethylene Glycols/therapeutic use[mesh] OR “polyethylene glycols” [tiab]))) AND (Tissue adhesions[mesh] OR adhes*[tiab])))) AND (randomized controlled trial [pt] OR random*)

Perioperative pharmacological and nutritional interventions with direct relation to the surgical procedure

Postoperative ileus management

P: Patients undergoing abdominal surgery
I: Alvimopan or Methylnaltrexone
C: Standard postoperative care (laxatives/ enema)
O: Mortality, morbidity, bowel movements, DGE

((((("alvimopan"[nm]) OR "alvimopan" [Supplementary Concept])) OR ADL 8-2698)) OR (((Alvimopan OR entereg)) OR (Methylnaltrexone OR Relistor))) AND (randomized controlled trial [pt] OR random*)

Pancreatic fistula or enteral fistula

P: Patients undergoing pancreatic surgery
I: Somatostatine
C: Placebo
O: Mortality, morbidity esp. POPF

P: Patients with enteral fistula
I: Somatostatine
C: Placebo
O: Mortality, morbidity, Fistula closure, Success of Operation

((("Octreotide"[Mesh] OR Octreotide [tiab] OR "Somatostatin"[Mesh] OR somatostatin [tiab] OR “somatostatin analogues” OR lanreotide [Substance Name] OR lanreotide [tiab] OR pasireotide [Substance Name] OR pasireotide [tiab] OR vapreotide [Substance Name] OR vapreotide [tiab] AND Pancreatectomy[MeSH] OR pancreatectomy [tiab] OR Pancreaticojejunostomy[MeSH] OR pancreaticojejunostomy [tiab] OR“pancreatico-jejunostomy” [tiab] OR Pancreaticoduodenectomy[MeSH] OR pancreaticoduodenectomy [tiab] OR pancreaticoduodenectomies [tiab] OR duodenopancreatectomy [tiab] OR duodenopancreatectomies [tiab] OR“pancreatic resection” [tiab] OR “pancreatic surgery”[tiab] OR “pancreatico-gastrostomy” OR pancreaticogastrostomy [tiab] OR Whipple) AND Randomized Controlled Trial[ptyp])) AND (randomized controlled trial [pt] OR random*)

Perioperative immunonutrition

P: Patients undergoing abdominal surgery
I: Perioperative immunonutrition
C: Standard nutrition
O: Mortality and morbidity

((((((((((((((((("perioperative immunonutrition") OR "postoperative immunonutrition")) OR (("Dietary Supplements"[Mesh]) OR “dietary supplements” [tiab])) OR ((“Enteral nutrition” [tiab]) OR “Enteral nutrition” [mesh])) OR Arginine [tiab]) OR “omega-3 fatty acid” [tiab]) OR Glutamine [tiab]) OR “diet supplementation” [tiab]) OR “oral supplement” [tiab]) OR “oral supplements” [tiab]) OR “parenteral nutrition” [tiab]) OR “nutritional support” [tiab]) OR “Parenteral nutrition” [tiab]) OR “enteric feeding” [tiab]) OR “diet therapy” [tiab])) AND (surgery [tiab] OR surgeries[tiab] OR surgic*[tiab]))) AND ((randomized controlled trial [pt] OR random*))
