# Supplementary material for: Association of industry sponsorship and positive outcome in randomised controlled trials in general and abdominal surgery: protocol for a systematic review and empirical study
Source: Syst Rev. 2014 Nov 27;3:138. doi: 10.1186/2046-4053-3-138 (PMC4280764; doi:10.1186/2046-4053-3-138)
Supplement: Supplementary file 2 — Additional file 2: Extraction sheet. Standardised data collection form that will be used to extract data from included studies. (PDF 289 KB) [file 13643_2014_313_MOESM2_ESM.pdf]

Study No.: *"#"* ***Topic:*** *"Mesh Preventive"*

Data Extractor: ☐ PP ☐ KG ☐ DATABASE

Characteristics **Title:**

Author: **Pub Year:** **Journal:**

Language ☐ **english** ☐ **other:**

Trial registered ☐ **no** ☐ **yes** **at:** **Registration number:**

Study duration **from** **to** ☐ **not stated**

Participating centres ☐ **mono-centre** ☐ **multicentre (No. of centres:)**

Participating Country ☐ **international:** ☐ **national:**

Number of treatment groups ☐ **two-arm** ☐ **multi-arm (No. of arms:)**

Sample size calculation **n=** **Power %** **Delta %**

Analysis strategy ☐ **ITT** ☐ **PP** ☐ **other:** ☐ **not stated**

Patients total **n=** **Patients evaluable** **n =**

Withdrawals **n =** **Lost to follow up** **n =**

CONFLICT OF INTEREST

COI ☐ **stated** ☐ **not stated**

If stated, how was COI described?

FUNDING ☐ **industry** ☐ **independent** ☐ **not stated**

Sponsor:

If industry, was it stated as COI? ☐ **no** ☐ **yes**

Number of authors affiliated with industry:

Type of industry support:

INTERVENTIONS

Operation

Surgical Standardization ☐ **standardized** ☐ **non-standardized** ☐ **not stated**

Intervention performed by ☐ **trained surgeons** ☐ **teaching-OP** ☐ **not stated**

Comparability at baseline ☐ **affirmed** ☐ **not affirmed**

Reason:

OUTCOMES

Number of outcomes **Primary:** **Secondary:**

Primary outcome defined

Outcome ☐ **Not-Pro** ☐ **Pro**

p-value of primary outcomes

Key conclusions of the study authors

Conclusions supported by data ☐ **no** ☐ **yes**

If "no", state:

RISK OF BIAS

Stratified randomization ☐ **no** ☐ **yes, strata:**

Block randomization ☐ **no** ☐ **yes, size of block:** ☐ **not stated**

Random sequence generation

- ☐ **computer generated random-numbers**
- ☐ **drawing envelopes**
- ☐ **table of random number list**
- ☐ **non-random approach**
- ☐ **other:**
- ☐ **unclear**
- ☐ **not stated**

Risk: ☐ **Low** ☐ **High** ☐ **Unclear**
Notes:

Allocation concealment

- ☐ **sequentially numbered, sealed, opaque envelopes**
- ☐ **central randomisation**
- ☐ **open random allocation schedule**
- ☐ **alternation or rotation**
- ☐ **other:**
- ☐ **unclear**
- ☐ **not stated**

Risk: ☐ **Low** ☐ **High** ☐ **Unclear**
Notes:

Blinding (I)

- ☐ **Patients**
- ☐ **Practitioners**
- ☐ **Data collectors**
- ☐ **not stated**

Risk: ☐ **Low** ☐ **High** ☐ **Unclear**
Notes:

Blinding (II)

☐ **Outcome assessors**
☐ **Data analysts**
☐ **not stated**

Risk: ☐ **Low** ☐ **High** ☐ **Unclear**
Notes:

Incomplete outcome data ☐ **no** ☐ **yes** ☐ **not stated**

Risk: ☐ **Low** ☐ **High** ☐ **Unclear**
Notes:

Selective reporting ☐ **no** ☐ **yes** ☐ **not stated**

Risk: ☐ **Low** ☐ **High** ☐ **Unclear**
Notes:

Other bias ☐ **no** ☐ **yes** ☐ **not stated**
Description:

Risk: ☐ **Low** ☐ **High** ☐ **Unclear**
Notes:

Important notes:

Date: .... **Signature:** ....
